# Complete Coding Sequences of Two Dengue Virus Type 2 Strains Isolated from an Outbreak in Burkina Faso in 2016

**DOI:** 10.1128/genomeA.00209-17

**Published:** 2017-04-27

**Authors:** Cécile Baronti, Géraldine Piorkowski, Franck Touret, Rémi Charrel, Xavier de Lamballerie, Antoine Nougairede

**Affiliations:** a UMR “Emergence des Pathologies Virales” (EPV) Aix-Marseille University, IRD 190, Inserm 1207, EHESP, Marseille, France; b Institut Hospitalo Universitaire Méditerranée-Infection, Marseille, France

## Abstract

We report here the complete coding sequences of two strains of dengue virus type 2, isolated in France from patients returning from Burkina Faso in November 2016. Both strains (cosmopolitan genotype) are almost identical (99.91% nucleotide identity) and closely related to a strain circulating in Burkina Faso in 1983.

## GENOME ANNOUNCEMENT

Dengue virus (DENV; family *Flaviviridae*, genus *Flavivirus*) is an arbovirus mainly transmitted by *Aedes* mosquitoes. There are four dengue serotypes (DENV-1 through DENV-4) that together are responsible each year for millions of cases of human infections ranging from acute febrile illness to life-threatening dengue hemorrhagic fever (1). Dengue is endemic in more than 100 countries in America, Asia, Oceania, and Africa (1). However, little is known about dengue in Africa due to poor surveillance and underrecognition of the disease (2). Dengue epidemics have been reported in Africa since the 19th century and for the first time in Burkina Faso in 1925 (3, 4).

In 2016, an outbreak of dengue was reported in Burkina Faso by the World Health Organization (5). In November 2016, two travelers returning from Ouagadougou were diagnosed as having noncomplicated dengue fever in Marseille, France: a 25-year-old woman [this case was previously described (6)] and a 28-year-old woman. DENV-2 RNA was detected by real-time reverse transcription-PCR (RT-PCR) in the serum of both patients (7). Two DENV-2 clinical strains, called 7869191/BF/2016 and 7754691/BF/2016, respectively, were isolated from sera using C6/36 cells. Both isolates are available to the scientific community via the European Virus Archive goes Global (EVAg) project, a nonprofit organization.

After three passages in C6/36 cells, viral RNA was extracted from cell culture supernatant. Overlapping RT-PCR products spanning the complete genome were generated using a specific set of primers previously described (8), purified, pooled, and sequenced using next-generation sequencing (Ion Torrent, Thermo Fisher Scientific). Around 80,000 reads were produced for each sample, from which two contigs with a length of 10,675 nucleotides (nt) were generated with CLC Genomics Workbench software (CLC bio).

Both genomes display a classical DENV-2 genome organization (9) and contain a unique open reading frame (10,176 nt) that encodes a polyprotein processed in three structural proteins—capsid (100 amino acids [aa]), premembrane/membrane (166 aa), and envelope (495 aa)—and seven nonstructural proteins—NS1 (352 aa), NS2A (218 aa), NS2B (130 aa), NS3 (618 aa), NS4A (127 aa), NS4B (248 aa), and NS5 (900 aa). Cleavage sites are identical to those previously reported (9).

As previously described, type 2 DENVs are divided into six genotypes based on nucleotide sequences of the envelope gene (10, 11). Phylogenetic analysis revealed that strains 7869191/BF/2016 and 7754691/BF/2016 belong to the cosmopolitan genotype that contains strains from Asia, Africa, and the Pacific. Their nucleotide and amino acid sequences are 99.91% identical (10,665/10,675 nt; 3388/3391 aa), demonstrating that both patients were infected by the same epidemic strain. Phylogenetic analysis also indicated that these clinical isolates shared a common ancestor with a DENV-2 strain circulating in Burkina Faso in 1983 (GenBank accession no. EU056810; 97.1% nt and 99.4% aa identity). This interesting relationship suggests that this strain of DENV-2 virus has been endemic in Burkina Faso for more than 30 years, probably maintained through a sylvatic (nonhuman primate/sylvatic mosquitoes) cycle.

These genome sequences might contribute to a better understanding of the molecular epidemiology of DENVs in Burkina Faso.

### Accession number(s).

The virus genome sequences described here have been deposited in GenBank under the accession numbers KY627762 and KY627763 for strains 7869191/BF/2016 and 7754691/BF/2016, respectively.
